# Cytotoxic Granule Exocytosis From Human Cytotoxic T Lymphocytes Is Mediated by VAMP7

**DOI:** 10.3389/fimmu.2019.01855

**Published:** 2019-08-07

**Authors:** Praneeth Chitirala, Keerthana Ravichandran, Donatella Galgano, Marwa Sleiman, Elmar Krause, Yenan T. Bryceson, Jens Rettig

**Affiliations:** ^1^Cellular Neurophysiology, Center for Integrative Physiology and Molecular Medicine (CIPMM), Saarland University, Homburg, Germany; ^2^Center for Hematology and Regenerative Medicine (HERM), Karolinska Institute, Stockholm, Sweden

**Keywords:** cytotoxic T lymphocytes, cytotoxic granules, SNARE proteins, immunological synapse, familial hemophagocytic lymphohistiocytosis, T cell killing, exocytosis, longin domain

## Abstract

Cytotoxic T lymphocytes kill infected or malignant cells through the directed release of cytotoxic substances at the site of target cell contact, the immunological synapse. While genetic association studies of genes predisposing to early-onset life-threatening hemophagocytic lymphohistiocytosis has identified components of the plasma membrane fusion machinery, the identity of the vesicular components remain enigmatic. Here, we identify VAMP7 as an essential component of the vesicular fusion machinery of primary, human T cells. VAMP7 co-localizes with granule markers throughout all stages of T cell maturation and simultaneously fuses with granule markers at the IS. Knock-down of VAMP7 expression significantly decreased the killing efficiency of T cells, without diminishing early T cell receptor signaling. VAMP7 exerts its function in a SNARE complex with Syntaxin11 and SNAP-23 on the plasma membrane. The identification of the minimal fusion machinery in T cells provides a starting point for the development of potential drugs in immunotherapy.

## Introduction

Cytotoxic T lymphocytes (CTLs) fulfill an important task by eradicating infected or neoplastic cells. T cell development involves somatic recombination of T cell receptor genes followed by a clonal selection process that eradicates self-reactive cells. Target cells are recognized and killed upon T cell receptor recognition of non-self peptides in the context of MHC class I molecules. Once an epitope is recognized, CTLs undergo a major rearrangement to form a transient contact site with the target cell, the immune synapse (IS). Cytotoxic granules (CGs), vesicles that contain cytotoxic material like perforin and granzymes, are transferred along the cytoskeleton toward the IS and release their material upon fusing with the plasma membrane. Because of the hazardous content of CGs, the fusion process is tightly regulated in space and time.

Vesicular fusion is mediated by “soluble NSF attachment receptor (SNARE)” proteins. These proteins reside on the target (plasma) membrane (tSNARE) and on the vesicle membrane (vSNARE) and form during fusion the so-called SNARE complex consisting of three α-helices contributed from tSNAREs and one α-helix contributed from the vSNARE (1–3). The human genome contains 35 SNARE proteins, 26 tSNAREs, and 9 vSNAREs (4). They are also instrumental in mediating CG fusion with the plasma membrane at the IS (5), because e.g., mutations in the vSNARE Syntaxin11 lead to familial hemophagocytic lymphohistiocytosis (HLH) type 4 (FHL4), a life-threatening immune disorder defined by the inability of CTLs to kill target cells (6–8). Furthermore, other familial forms of HLH are caused by mutations in genes encoding perforin, a cytotoxic granule constituent required for target cell killing, as well as Munc13-4 and Munc18-2, representing key regulators of SNARE complex assembly and membrane fusion. While it seems established that Syntaxin11 and SNAP-23 are the tSNAREs mediating CG fusion in human CTLs, the identity of the vSNARE remains enigmatic.

Analyzing transcription and protein expression, it was shown that primary, activated CTLs from human blood express at least 7 vSNAREs (9). We have tested several vSNAREs for their role in CG fusion and identify by tetanus toxin treatment, immunocytochemistry, and siRNA-mediated knock-down experiments. Our results demonstrate that VAMP7 (aka TI-VAMP) is the major vSNARE responsible for mediating the final fusion step of CGs at the IS of human CTLs.

## Materials and Methods

### Cells

The local ethics committee has approved research with human material performed for this study. Human peripheral blood mononuclear cells (PBMCs) were isolated from 3 to 5 anonymous healthy donors per experiment (N) by density gradient centrifugation (Lymphoprep; Axis-Shield). In some experiments, PBMCs were stimulated with 5 μg/ml of SEA at a density of 10^8^ cells/ml at 37°C for 1 h. Stimulated PBMCs were thereafter resuspended at a density of 1.5 × 10^6^ cells/ml in complete medium (AIMV medium; Thermo Fisher Scientific) supplemented with 10% FCS (Thermo Fisher Scientific) supplemented with 100 IU/ml of recombinant human IL-2 (Biosource). After 5 days, SEA-specific CTLs were positively selected (Dynabeads CD8 positive isolation kit; Thermo Fisher Scientific) and cultured further in complete medium. CTLs from day 1 to 2 after positive isolation were used for experiments. For bead-stimulated CTLs, CD8+ T cells were isolated from PBMCs by negative magnetic selection (Dynabeads untouched CD8 T cell kit; Thermo Fisher Scientific) and stimulated with antibody-coated beads (Dynabeads Human T-Activator CD3/CD28; Thermo Fisher Scientific) in complete medium.

### Antibodies

For Western blotting, anti-phospho-p42/44 MAPK (Erk1/2, Thr202/Tyr204; 9101), anti-GAPDH (14C10, 2118L), anti-VAMP2 (D6O1A, 13508), anti-FLAG (2368; all from Cell Signaling Technology), anti-LAT (06-807; Merck), anti-SNAP 23 (111203; Synaptic Systems), anti-Syntaxin 11 (110113; Synaptic Systems), anti-VAMP7 (NBP2-32232; Novus Biologicals), and HRP-conjugated anti-Strep-tagII (2-1509-001; IBA Lifesciences) antibodies were used. Secondary antibodies were HRP-conjugated donkey anti-rabbit (Thermo Fisher Scientific). For Structured Illumination Microscopy (SIM), Alexa Fluor 647 anti-human Perforin (DG9; BioLegend), and anti-granzyme B (GB11; BioLegend) antibodies were used. For Total Internal Reflection Microscopy (TIRF) monoclonal mouse anti-human anti-CD3ε (B-B11; Diaclone), monoclonal mouse anti-human CD28 (CD28.2; BD) antibodies were used for coating coverslips and stimulating cells.

### Plasmids and siRNA

VAMP3, VAMP4, VAMP7, and VAMP8 constructs were purchased from Addgene (42310, 42313, 42316, and 42311). VAMP7 coding sequence was amplified from pEGFP-VAMP7 plasmid (42316; Addgene) with forward primer 5′-ATA TAC GGG GTA CCG CCG CCA CCA TGG CGA TTC TTT TTG CT-3′ and reverse primer 5′-ATA TAC CGG AAT TCT TTC TTC ACA CAG CTT GG-3′ and inserted in frame with C-terminal mCherry with forward primer 5′-ATA TAC CCA AGC TTA TGG TGA GCA AGG GCG AG-3′ and reverse primer 5′-ATA TAC GCG GAT CCT TAC TTG TAC AGC TCG TCC AT-3′ or inserted in frame with pHuji with forward primer 5′-ATG TAT ACC CAA GCT TAT GGT GAG CAA GGG CGA G-3′ and reverse primer 5′-ATG TAT ACG CGG ATC CTT ACT TGT ACA GCT CGT C-3′ in pMAX containing GGSGGSGGS linker. Similarly, human VAMP8-mCherry, granzyme B-mCherry, granzyme B-mTFP, and msVAMP2-mRFP constructs have been previously described (10, 11). Control siRNA: 5′-UUC UCC GAA CGU GUC ACG UTT-3′, VAMP7 siRNA 1; 5′-GAU UGG AAU UAU UGA UUG ATT-3′, VAMP7 siRNA 2: 5′-GGG CAA UCG UGU CGC UAA UTT-3′, VAMP4 siRNA 1: 5′-AGA UUG CUG CAU AAU UUA ATT-3′ were purchased from Qiagen.

### Semi-quantitative PCR

Total RNA was extracted with TRIzol (Invitrogen) and reverse transcribed (SuperScript II; Thermo Fisher Scientific) using random hexamer primers. Semi-quantitative PCR was performed using specific VAMP4 forward primer 5′-CCT TCG AAG TTG TTT GGA TC-3′ and reverse primer 5′-GGA CCA AGA TTT GGA CCT AG-3′. GAPDH was used as loading control using forward primer 5′-ACC ACA GTC CAT GCC ATC AC-3′ and reverse primer 5′-TCC ACC ACC CTG TTG CTG TA-3′. Data were normalized to GAPDH.

### Western Blot Analysis

Human CTLs were homogenized with a syringe in lysis buffer (50 mM Tris (pH 7.4), 1 mM EDTA, 1% Triton X-100, 150 mM NaCl, 1 mM DTT, 1 mM deoxycholate, protease inhibitors, and PhosSTOP; Roche) on ice. Lysates were rotated for 10 min at 4°C, and insoluble material was removed by centrifugation at 13,000 RPM. The protein concentration was determined using Quick Start Bradford 1x Dye Reagent (5000205; Bio-Rad). Proteins were separated by SDS-PAGE (NuPAGE; Thermo Fisher Scientific), transferred to nitrocellulose membranes (Amersham), and blocked by incubation with 5% skim milk powder in 20 mM Tris, 0.15 M NaCl, pH 7.4, and TBS for 1–2 h and blotted with specific antibodies. Blots were developed using enhanced chemiluminescence reagents (SuperSignal West Dura Chemoluminescent Substrate; Thermo Fisher Scientific) and scanned. For expression analysis, the pixel area and mean fluorescence intensity (MFI) were determined with Fiji v1.46 (12).

### Nucleofection of Expression Constructs and siRNA-Mediated Knockdown

After 2 days of bead stimulation, 5 × 10^6^ CTLs were transfected with 1–2 μg of plasmid DNA (P3 Primary Cell 4D-Nucleofector X Kit, V4XP-3024; Lonza) Cells were imaged 10–12 h after transfection. For knockdown of protein expression, 3–5 × 10^6^ CTLs were transfected with 2 μM of siRNA (Hs_SYBL1_9 and Hs_SYBL1_7, SI04376134, and SI04212453; QIAGEN) using the Nucleofector kit (Lonza). After 6 h of incubation, cells were washed and cultured in AIM V media with 10% FCS and used for experiments 12–18 h after transfection. Knockdown efficiency was assessed by preparing whole cell lysates of 10^6^ CTLs boiled in SDS loading buffer containing 4% β-mercaptoethanol and 1 mM DTT. Lysates were sonicated, proteins were separated by SDS-PAGE, and expression was analyzed by Western blotting.

### Structured Illumination Microscopy (SIM)

To conjugate CTLs with target cells, Raji cells (ATCC, CCL-86) were pulsed with 10 μg/ml SEA at 37°C for 30 min. The stimulation of Raji cells was performed in 96-well plates with a maximum of one million cells resuspended in 100 μl AIM V medium. The CTLs and SEA pulsed Raji cells were washed once with AIM V and resuspended at a concentration of 2 × 10^6^ cells/ml. CTLs were mixed with target cells at a 10:1 ratio and plated onto poly-L-ornithine coated or CD3ε coated 12 or 25 mm glass coverslips and incubated at 37°C for 5, 10, and 15 min. Cells were fixed in ice-cold 4% PFA in Dulbecco's 1xPBS (Thermo Fisher Scientific) and stained with antibodies. The SIM setup was an Elyra PS1 System (Carl Zeiss Microscopy, GmbH, Jena). Images were acquired with a 63x Plan-Apochromat (NA 1.4) with laser excitation at 488, 561, and 635 nm and then processed to obtain higher resolutions (Zen 2012; Carl Zeiss, Jens). For analysis of colocalization, Pearson's and Manders' overlap coefficients were determined using the JACoP plugin of Fiji v1.46 (12).

### Total Internal Reflection Fluorescence (TIRF) Microscopy

The TIRFM setup from Visitron Systems GmbH (Puchheim, Germany) was based on an IX83 (Olympus) equipped with the Olympus autofocus module, a UAPON100XOTIRF NA 1.49 objective (Olympus), a 488 nm 100 mW laser and a solid-state laser 100 mW emitting at 561 nm, the iLAS2 illumination control system (Roper Scientific SAS, France), the evolve-EM 515 camera (Photometrics) and a filter cube containing Semrock (Rochester, NY, USA) FF444/520/590/Di01 dichroic and FF01-465/537/623 emission filter. The setup was controlled by Visiview software (Version:4.0.0.11, Visitron GmbH). CTLs from 2 different donors were electroporated with a GzmB-mTFP plasmid as a CG marker to image fusion in TIRFM as described before (11). For other experiments CTLs were transfected with GzmB-mCherry or VAMP7-mCherry or VAMP7-pHuji or rab11-mCherry plasmids. After 12–16 h of transfection, CTLs (0.2–0.3 × 10^6^ cells) were resuspended in 30 μl of extracellular buffer (2 mM HEPES, 140 mM NaCl, 4.5 mM KCl, and 2 mM MgCl_2_) containing no Ca^2+^ and allowed to settle for 1–2 min on anti-CD3ε antibody (30 μg/ml) coated coverslips. Cells were then perfused with extracellular buffer containing 10 mM Ca^2+^ for cytotoxic granule fusion. Depending on the constructs used and the experimental conditions, cells were imaged for 7 min at room temperature (RT = 22 ± 2°C) either at 561 nm or at 488 nm or alternating between both illuminations. Unless specified otherwise, acquisition frequency was 10 Hz, and the exposure time was 100 ms. Cytotoxic granule fusion analysis, images, and time-lapse series were analyzed using Fiji v1.46 (12). A sudden rise in VAMP7-pHuji fluorescence or a sudden drop in VAMP7-mCherry, GzmB-mTFP, GzmB-mCherry, or Rab11-mCherry fluorescence occurring within 300 ms (three acquisition frames) was defined as fusion (13). The number of vesicles was shown according to the corresponding fluorescence intensity of the vesicles.

### Calcein Killing Assay

For the population killing assay, Raji cells were pulsed with SEA (1 μg/ ml) for 30 min at 37°C and later cells were loaded with calcein-AM (500 nM; Life Technology) in serum-free IMDM for 15 min at RT. Cells were washed with PBS once and plated into 96-well black plates with clear bottoms (BD Falcon). Target cells were lysed with Triton X-100 (0.1%) to calculate maximum target cell lysis. CTLs were transfected with ns-siRNA or siRNA 1 or 2 against VAMP7 and, 16–18 h after transfection, added to target cells (10:1 ratio; 0.2 × 10^6^ cells/well) to measure killing at 37°C for 4 h. Readings were measured at 485 nm excitation wavelength and 535 nm emission wavelength by GENios Pro plate reader (Tecan Group AG). The fluorescence for the experimental conditions was adjusted by the parameter γ according to the live target cell control fluorescence. The γ value was measured at time zero: γ = F_live(0)_/F_exp(0)_. The cytotoxicity was calculated from the loss of calcein fluorescence in target cells using the following equation: % target cell lysis = (F_live_-γ × F_exp_)/(F_live_-F_lyse_) × 100%. Abbreviations in the equation are: fluorescence of only target cell controls (F_live_), CTLs with target cells (F_exp_), and maximum target lysis (F_lyse_). All experiments were performed in duplicates from 4 donors.

### Assessment of ERK Phosphorylation

Primary human CD8+ T cells (1 × 10^6^/ml) were activated for the appropriate time with anti-CD3ε (125 ng/ml) and anti-CD28 (250 ng/ml) in a volume of 1 ml. Cells were washed in cold PBS and lysed in lysis buffer [50 mM Tris (pH 7.4), 1 mM EDTA, 1% Triton X-100, 150 mM NaCl, 1 mM DTT, 1 mM deoxycholate, protease inhibitors, and PhosSTOP; Roche] on ice. Lysates were sonicated, proteins were separated by SDS-PAGE, and expression was analyzed by Western blotting.

### Immunoprecipitation (Twin-Strep-tag Pulldown Assay)

50 × 10^6^ of primary, human CD8+ T cells were transfected with either Twin-Strep-tag (control), VAMP7-Flag-Twin-Strep-tag, or Twin-Strep-tag-Stx11 and incubated at 37°C for 16 h. Cells were then lysed in ice-old lysis buffer [50 mM Tris (pH 7.4), 300 mM NaCl, 0.5% Triton X-100, protease inhibitor mix (Roche)] and centrifuged at 13,000 rpm for 10 min at 4°C. Following protein quantification, 500 μg of cleared lysate was incubated with 125 μg Strep-Tactin-Sepharose (IBA Lifesciences) for 1.5 h at 4°C. After pulldown, beads were washed three times with lysis buffer. Bound proteins were eluted with 1x NuPAGE^TM^ LDS sample buffer (Thermo Fisher Scientific) and heated at 98°C for 15 min for Western blot analysis. 50 μg (10%) of protein lysate was loaded as input.

### Statistics

Statistical differences in data were analyzed with paired or unpaired Student's *t*-test, and graphed using Affinity Designer Software (Serif Ltd).

## Results

### Fusion of Cytotoxic Granules Is Insensitive to Tetanus Toxin

**Figure 1:**
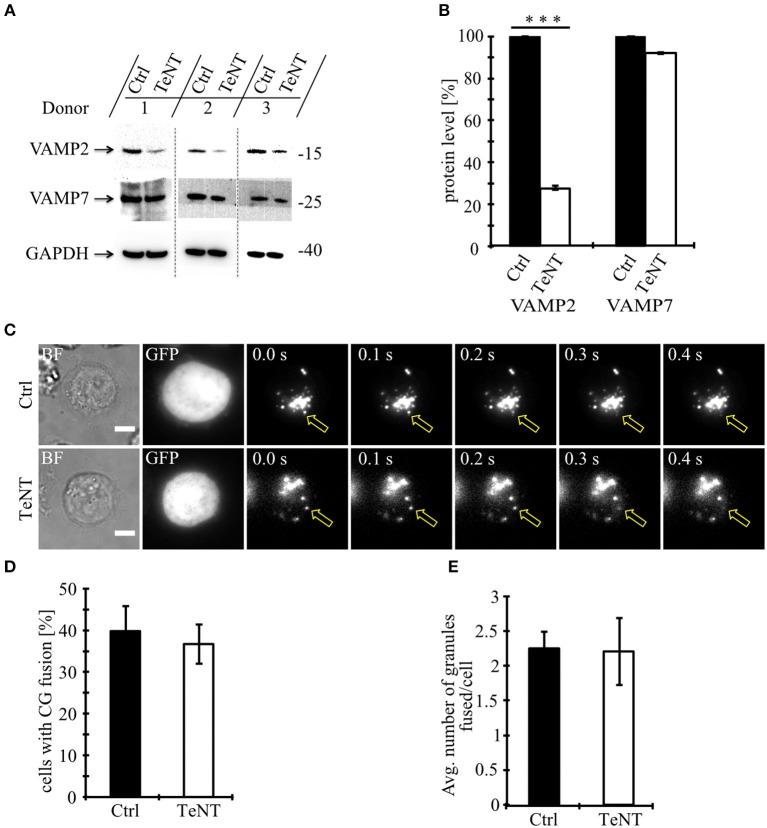
Fusion of cytotoxic granules with the plasma membrane is insensitive to Tetanus toxin. **(A)** Bead-stimulated human CD8+ T cells transfected with GFP or TeNT-GFP as indicated. VAMP2 and VAMP7 protein levels were determined by Western blot analysis 12–16 h after transfection. **(B)** Expression of VAMP2 and VAMP7 protein levels relative to GAPDH in CTLs transfected with GFP or TeNT-GFP as indicated. Graphs represent means [*N* = 3, ^***^p < 0.001 (Student's *t*-test)]. **(C)** Bead-stimulated human CTLs co-transfected with either GFP or TeNT-GFP along with granzyme B-mCherry and imaged 12 h after transfection. Representative live-cell TIRFM images of CTLs in contact with an anti-CD3 coated coverslip. Fusion events indicated with open arrowheads. **(D)** Mean percentage of cytotoxic granule fusion in cells transfected with either GFP (*n* = 50) or TeNT-GFP (*n* = 40), *p* = 0.704 (Student's *t*-test). **(E)** Mean average number of granules fused per cell over time in the TIRF plane in cells transfected with either GFP (*n* = 20) or TeNT-GFP (*n* = 15), *p* = 0.939 (Student's *t*-test). Bars show mean ± SEM. Scale bar, 5 μm.

We first used tetanus toxin as a functional screen to narrow the number of vSNAREs (VAMPs) that might be responsible for CG fusion. Tetanus toxin (TeNT) from the anaerobic *Clostridia* bacteria selectively cleaves VAMP1, VAMP2 (aka synaptobrevin2), and VAMP3 (aka cellubrevin), but not VAMP4, VAMP7 (aka TI-VAMP) (14), and VAMP8 (15, 16). We expressed the protease-containing light chain of TeNT in primary, human T lymphocytes and tested its functionality by Western blot comparing the expression levels of VAMP2 and VAMP7 (Figure 1A). As expected, TeNT expression resulted in efficient cleavage of VAMP2 (27.6 ± 1.3% expression) compared to CTLs expressing only GFP while the protein level of VAMP7 was unaffected (92.1 ± 0.9%; Figure 1B). We then performed total internal reflection fluorescence microscopy (TIRFM) to visualize and quantify fusion of individual CGs upon TeNT expression (Figure 1C). The percentage of CTLs showing fusion events was similar between CTLs expressing TeNT and CTLs expressing only GFP (Figure 1D). Similarly, the average number of granules that fused at the IS was similar between both groups (2.2 ± 0.5 for TeNT and 2.3 ± 0.4 for GFP; Figure 1E; Video 1). From these data we conclude that CG secretion from primary, human CTLs is insensitive to TeNT, eliminating VAMP1, VAMP2, and VAMP3 as candidates for mediating CG exocytosis.

### VAMP7 Shows a High Degree of Co-localization With Cytotoxic Granules, but Not With LAT-Containing Vesicles

**Figure 2:**
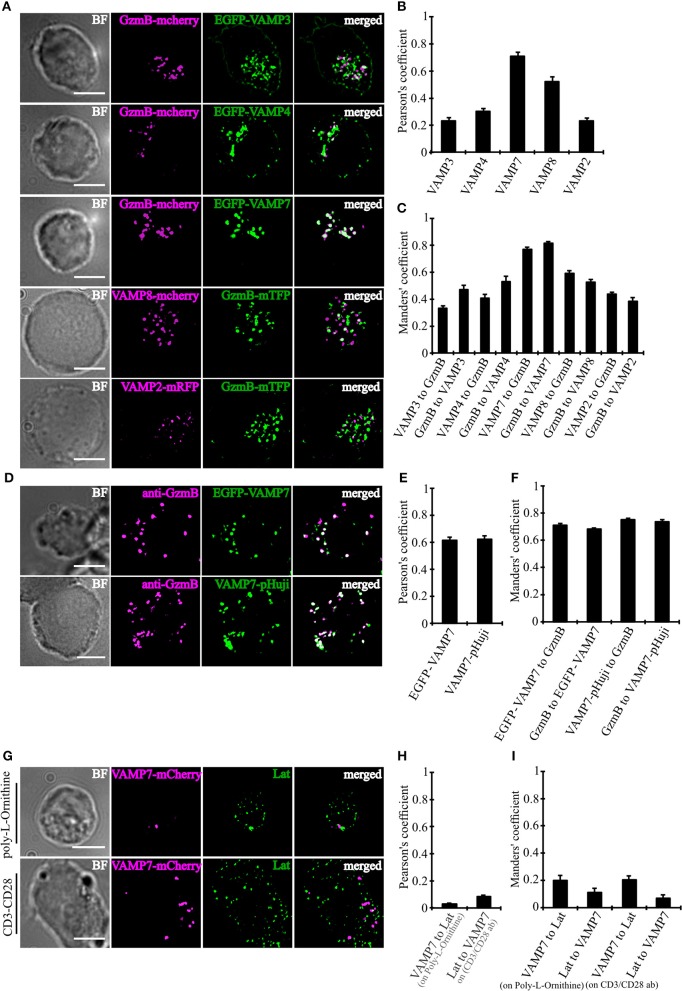
VAMP7 shows a high degree of co-localization with cytotoxic granules. **(A)** SIM images of bead-stimulated human CD8+ T cells co-transfected with either granzyme B-mCherry or granzyme B-mTFP along with the v-SNAREs mVAMP2, VAMP3, VAMP4, VAMP7, and VAMP8 constructs. Overlay of both channels displayed that only VAMP7 co-localizes with the lytic granule marker granzyme B. **(B)** Pearson's and **(C)** Manders' overlap coefficients for co-localization of VAMP3 (*n* = 16), VAMP4 (*n* = 14), VAMP7 (*n* = 13), VAMP8 (*n* = 9), and VAMP2 (*n* = 14) with granzyme B are given in the text. **(D)** Bead-stimulated human CD8+ T cells transfected with EGFP-VAMP7 or VAMP7-pHuji and immunolabeled with Alexa647-conjugated anti-granzyme B antibody. **(E)** The corresponding Pearson's and **(F)** Manders' overlap coefficients for colocalization of EGFP-VAMP7 (*n* = 12) or VAMP7-pHuji (*n* = 11) with granzyme B are given in the text. Data are shown as mean ± SEM. Scale bar, 5 μm. **(G)** Anti CD3/CD28 bead-stimulated human CD8+ T cells transfected with VAMP7-mCherry and incubated on either poly-L-ornithine or anti CD3 antibody coated coverslips for 15 min, fixed and immunolabeled with LAT antibody. **(H)** The corresponding Pearson's and **(I)** Manders' overlap coefficients for colocalization of VAMP7-mCherry with LAT on Poly-L-Ornithine coated coverslips (*n* = 11) or anti CD3/CD28 antibody coated coverslips (*n* = 10) are given in the text. Data are shown as mean ± SEM. Scale bar, 5 μm.

In order to determine which of the TeNT-insensitive VAMPs localize to CGs, we performed super-resolution structured illumination microscopy (SIM, 100 nm resolution in x, y, and 250 nm in z). Because only few paralog-specific antibodies for vSNAREs are available, we expressed fusion proteins of VAMP4, VAMP7, and VAMP8 together with a fusion protein of the CG marker granzyme B (GzmB) in primary CTLs derived from human blood (Figure 2A). From the three TeNT-insensitive VAMP paralogs tested, VAMP7 showed by far the highest Pearson's coefficient of correlation (17) and Manders' overlap coefficient (18). In contrast, VAMP4 showed only little overlap with the CG marker, resulting in Pearson's and Manders' values comparable to the TeNT-sensitive VAMP2 and VAMP3 that were used as controls (Figures 2B,C). VAMP8, which was shown previously to be responsible for the fusion of Rab11-positive recycling endosomes with the IS/plasma membrane prior to CG fusion (10), had an intermediate Pearson's coefficient of correlation (but see **Figures 4D–G**). Because in practice only values above 0.5 are considered a reasonable co-localization (17), we can conclude that VAMP7 is the most likely candidate for mediating CG fusion with the plasma membrane. This conclusion is supported by the immunocytochemical detection of GzmB with an anti-granzyme B-specific antibody that also showed a high degree of co-localization with expressed EGFP-VAMP7 and VAMP7-pHuji constructs (Figures 2D–F) and by Western blot data that demonstrate that VAMP7 protein levels are up-regulated upon activation of CTLs while VAMP2 protein levels are down-regulated (Figure S1).

In CD4+ T cells, VAMP7 plays an important role in the initial phase of IS formation by regulating the recruitment and phosphorylation of LAT, a key adaptor signaling protein required for T cell activation (19). In confocal and TIRF microscopy it was shown that LAT and VAMP7 partially co-localize on vesicles in close proximity to the IS. We therefore also examined LAT and VAMP7 localization in CD8+ T cells by super-resolution SIM in order to determine potential co-localization. However, in our experimental conditions we found basically no co-localization of LAT and VAMP7 (Figures 2G–I), ruling out that LAT is also localized on CGs.

### VAMP7 Polarizes to the IS on the Same Granules as Granzyme B and Perforin

**Figure 3:**
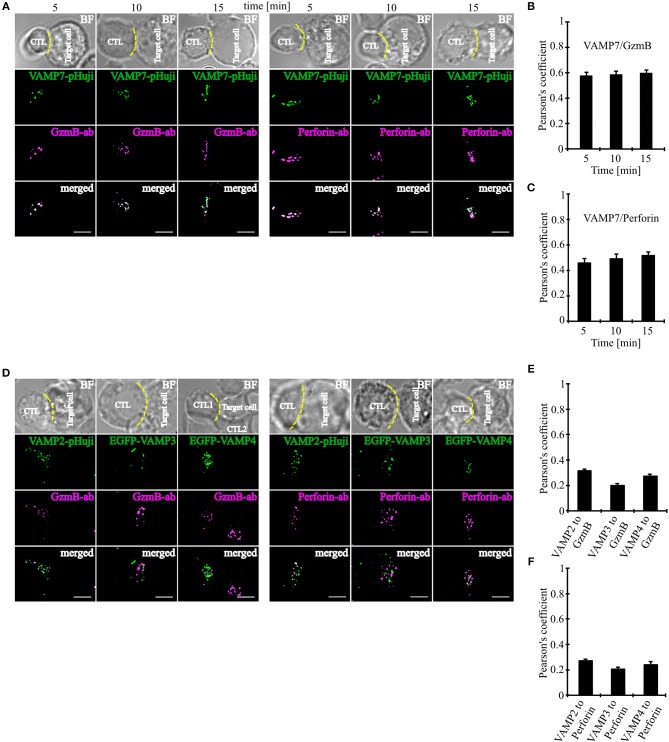
VAMP7 polarizes to the IS on the same granules as granzyme B and perforin. **(A)** SIM images of SEA-pulsed human CD8+ T cells transfected with VAMP7-pHuji construct and conjugated with SEA pulsed RAJI cells and fixed after 5, 10, and 15 min, respectively. CTLs were permeabilized and immunolabeled with either Alexa647-conjugated granzyme B or Alexa647-conjugated perforin antibody. **(B,C)** The corresponding Pearson's overlap coefficients for co-localization of VAMP7-mCherry with granzyme B (*n* = 12) and VAMP7-pHuji with perforin (*n* = 11) are given in the text. Data are shown as mean ± SEM. Scale bar, 5 μm. **(D)** SIM images of SEA-pulsed human CD8+ T cells transfected with VAMP2-pHuji, EGFP-VAMP3, and EGFP-VAMP4 constructs and conjugated with SEA pulsed RAJI cells and fixed after 15 min. CTLs were permeabilized and immunolabeled with either Alexa647-conjugated granzyme B or Alexa647-conjugated perforin antibody. **(E,F)** The corresponding Pearson's overlap coefficients for co-localization of VAMP2, VAMP3, and VAMP4 with granzyme B (*n* = 15) or perforin (*n* = 16) are given in the text. Data are shown as mean ± SEM. Scale bar, 5 μm.

Our initial co-localization studies were performed on activated human CTLs which were not in contact with target cells. To investigate whether VAMP7 co-localizes with CG markers during IS formation, we performed super-resolution SIM microscopy experiments after transfecting the cells with a VAMP7-pHuji fusion construct. 12 h after transfection CTLs were incubated with Raji target cells for 5, 10, and 15 min. Following fixation cells were stained with anti-granzyme B (Figure 3A left) or anti-perforin antibody (Figure 3A right), respectively. We found that CTLs in contact with target cells showed, as expected, a strong polarization of CGs toward the IS. Importantly, in both cases we found a highly significant co-localization of VAMP7 with the CG marker proteins at any time point measured, with Pearson's coefficients of correlation of 0.58 ± 0.03 (5 min), 0.59 ± 0.03 (10 min), and 0.60 ± 0.02 (15 min) for granzyme B (Figure 3B) and 0.46 ± 0.03 (5 min), 0.49 ± 0.03 (10 min), and 0.52 ± 0.03 (15 min) for perforin (Figure 3C), respectively. In contrast, VAMP2, VAMP3, and VAMP4 did not co-localize with granzyme B or perforin 15 min after contact with target cells (Figures 3D–F).

### VAMP7 Fuses at the Plasma Membrane Along With Granzyme B

**Figure 4:**
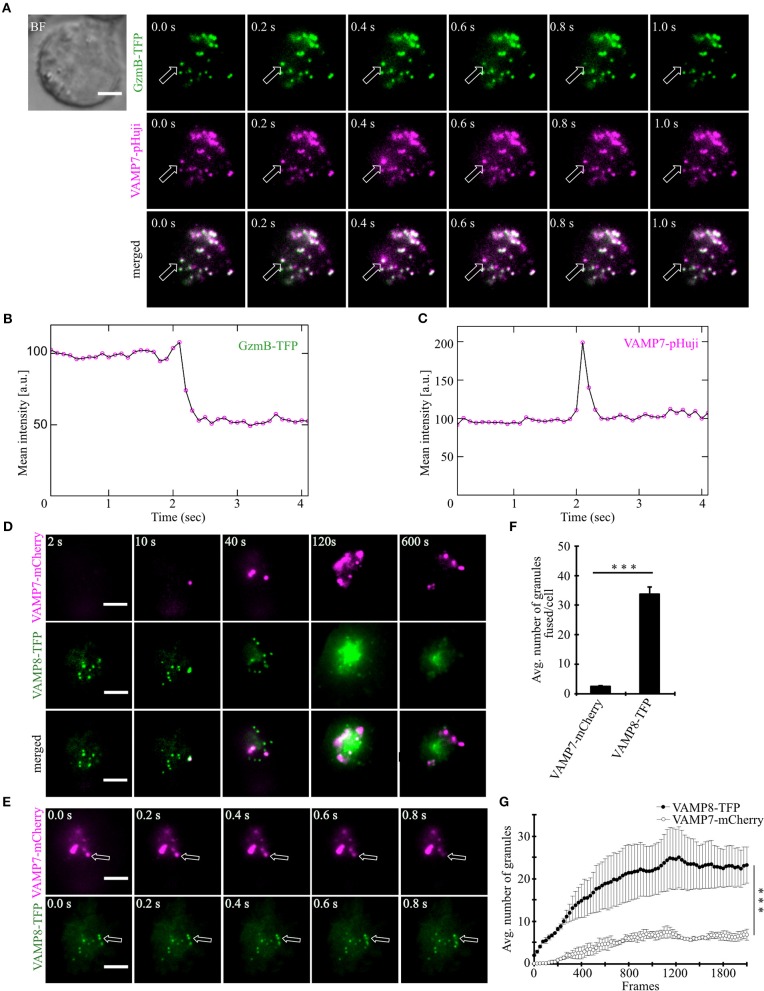
VAMP7 fuses at the plasma membrane along with granzyme B while VAMP8 fuses earlier and more frequently. **(A)** Bead stimulated human CTLs co-transfected with granzyme B-mTFP and VAMP7-pHuji constructs and imaged 12 h after transfection. Selected live-cell TIRFM images of granzyme B-mTFP and VAMP7-pHuji in a transfected CTL in contact with an anti-CD3 coated coverslip (GzmB, green, upper panel; VAMP7, magenta, middle panel; merged, lower panel). **(B,C)** Graphs depict the mean fluorescence intensity of individual mTFP and pHuji vesicles, respectively, over time. Experiments were repeated with three individual donors (*n* = 15 cells). Scale bar, 5 μm. **(D)** Bead stimulated human CTLs co-transfected with VAMP7-mCherry and VAMP8-mTFP constructs and imaged 12 h after transfection. Selected live-cell TIRFM images of VAMP7-mCherry and VAMP8-mTFP in a transfected CTL in contact with an anti-CD3 coated coverslip (VAMP7, magenta, upper panel; VAMP8, green, middle panel; merged, lower panel). **(E)** Exemplary, individual fusion events (arrows) of VAMP7- (upper panel) and VAMP8-containing vesicles (lower panel) at the indicated time of recording. Acquisition frequency 5 Hz. **(F)** Mean cumulative fusion events in the TIRF plane per cell [*N* = 3, *n* = 39, ^***^p < 0.001 (*t*-test)]. **(G)** Mean average number of VAMP7- and VAMP8-containing vesicles in the TIRF plane per cell in the first 3.5 min of measurements [*N* = 3, *n* = 39, ^***^p < 0.001 (Mann-Whitney *U*-test)]. Data are shown as mean ± SEM. Scale bar, 5 μm.

After demonstrating that VAMP7 is localized on CGs in activated as well as target cell-conjugated CTLs, we carried out TIRFM experiments with high temporal resolution (10 Hz) to test whether fusion of VAMP7 occurs simultaneously with the release of cytotoxic substances from CGs. Human CTLs were co-transfected with granzyme B coupled to mTFP (monomeric teal fluorescent protein) (20) and VAMP7 coupled to the pH-sensitive fluorophore pHuji (21). After seeding CTLs on CD3-coated coverslips the IS was formed quickly and we observed a fast accumulation of CGs, indicated by individual green (GzmB-mTFP) and magenta (VAMP7-pHuji) puncta (Figure 4A upper panels). In agreement with the data in Figures 2, 3, the merged image showed an almost complete co-localization of VAMP7 with the CG marker granzyme B (Figure 4A lower panel; Video 2). Individual fusion events were identified by a sudden drop in the mean intensity of GzmB-mTFP fluorescence (Figure 4B; Video 2), caused by the release and subsequent diffusion of the soluble GzmB-mTFP (compare frame 3 and 4 in the upper panel of Figure 4A). In parallel, we observed a sharp increase in VAMP7-pHuji fluorescence, caused by the opening of the fusion pore and subsequent dequenching of the luminal fluorophore (Figure 4A; exemplary trace in Figure 4C; Video 2). As expected, the magenta fluorescence persisted after fusion, because any vSNARE localized on the CG will remain in the plasma membrane after fusion (until endocytosis occurs) (22). Qualitatively similar results were obtained upon co-transfection of CTLs with VAMP7-mCherry and GzmB-mTFP (Figure S2). These data not only show that VAMP7 is selectively localized on CGs immediately prior to their fusion, but also verify that the protein is integrated into the CG membrane in the correct orientation. Because VAMP8 has been speculated to be involved in CG exocytosis (23) as well and showed an intermediate Pearson's coefficient of correlation (Figure 2), we performed TIRF experiments upon co-expression of VAMP7-mCherry and VAMP8-mTFP. If VAMP8 would be involved in the final fusion step of CGs with the plasma membrane (24, 25), we would have expected a co-localization of both VAMP isoforms. In agreement with the co-localization data of GzmB with different VAMP isoforms (Figures 2A–C) and our previous report (10), while both VAMP7 and VAMP8 were localized on vesicles, they showed virtually no co-localization at the IS. VAMP8-containing vesicles arrived earlier at the IS (Figure 4D; Video 3) and fused earlier and with 10-fold higher frequency than VAMP7-containing vesicles (Figures 4E–G; Video 3), again confirming our earlier findings (10) and essentially ruling out a role for VAMP8 in CG fusion with the plasma membrane.

### Knockdown of VAMP7 Strongly Reduces Fusion of Cytotoxic Granules at the IS

**Figure 5:**
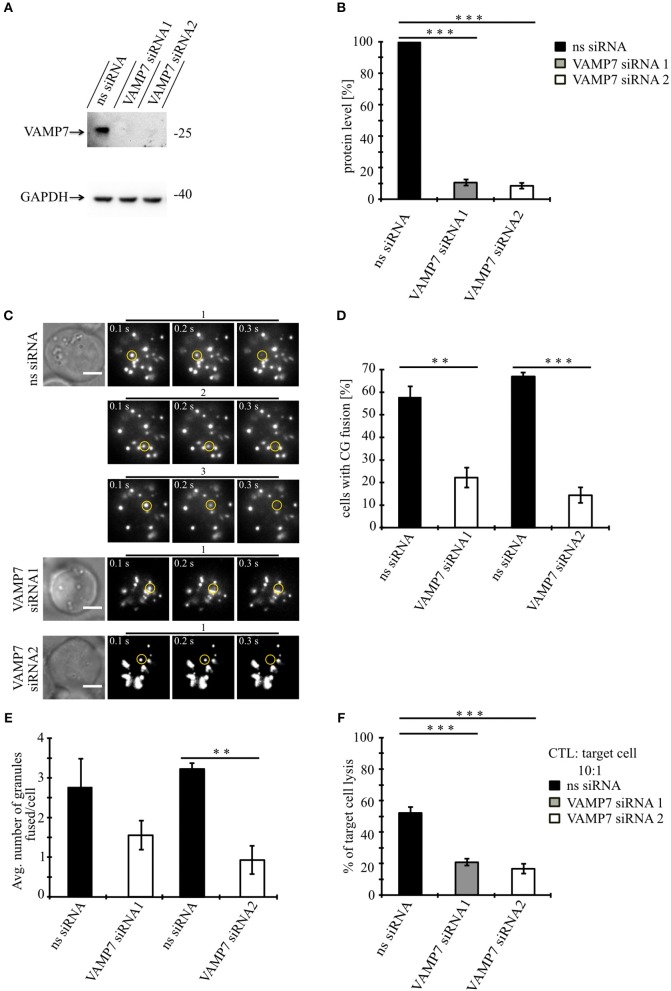
Knockdown of VAMP7 strongly reduces fusion of cytotoxic granules at the IS. **(A)** Lysates from bead stimulated human CD8+ T cells transfected with either control or VAMP7 siRNAs (1 or 2, respectively) and blotted for VAMP7 (top) and GAPDH (bottom) as loading control. **(B)** Quantification of VAMP7 protein expression (in % normalized to control siRNA-treated CTLs) performed by densitometry. Bars indicate SEMs. [VAMP7-siRNA1, *N* = 3; ^***^p < 0.001 and VAMP7-siRNA2, *N* = 3; ^***^p < 0.001 (*t*-test)]. **(C)** Human CD8+ T cells co-transfected with granzyme B-mCherry along with either ns-siRNA or VAMP7-siRNA1 or VAMP7-siRNA2 and imaged 12 h after transfection. Selected live-cell TIRF microscopy images of granzyme B-mCherry in a transfected CTL in contact with an anti-CD3 coated coverslip. Fusion events are indicated with open circles (three frames shown per granule fused). **(D)** Mean percentage of cytotoxic granule fusion in cells transfected with either ns-siRNA (*n* = 66 and *n* = 59, respectively) or VAMP7-siRNA1 [*n* = 91; ^**^p < 0.01 (*t*-test)] or VAMP7-siRNA2 [*n* = 72; ^***^p < 0.001 (*t*-test)]. **(E)** Mean average number of granules fused over time in the TIRF plane per cell *p* = 0.206 (*t*-test) for VAMP7-siRNA1 and ^**^p < 0.01 (*t*-test) for VAMP7-siRNA2. Bars indicate mean ± SEM. Scale bar, 5 μm. **(F)** Calcein-based killing assay for CTLs transfected with either ns-siRNA, VAMP7-siRNA1, or VAMP7-siRNA2. Experiments were carried out in duplicate [*N* = 4; ^***^p < 0.001 (*t*-test)].

Our data so far demonstrate that VAMP7 co-localizes with the CG markers granzyme B and perforin until CGs fuse with the plasma membrane at the IS. To investigate whether VAMP7 is functionally involved in the fusion of CGs with the plasma membrane, we performed TIRFM experiments following the knockdown of VAMP7 protein. For that purpose, we transfected primary human CTLs with two different siRNAs against VAMP7 together with GzmB-mCherry to visualize CGs. In contrast to a scrambled control siRNA, either VAMP7 siRNA reduced the expression of VAMP7 to 10.5 ± 3.9% (VAMP7 siRNA#1; ^***^p < 0.001) and 8.5 ± 1.9% (VAMP7 siRNA#2; ^***^p < 0.001) of the original level, respectively (Figures 5A,B). Both siRNAs significantly reduced the number of CTLs (Figures 5C,D), showing CG fusion to <50% of the values measured upon transfection of control siRNA (from 57.8 ± 4.8% to 22.2 ± 4.4% for VAMP7 siRNA#1; ^**^p < 0.01; and from 67.1 ± 1.8% to 14.4 ± 3.4% for VAMP7 siRNA#2, respectively; ^***^p < 0.001; Figure 5D). Simultaneously, the average number of CG fusion events observed in CTLs expressing either VAMP7 siRNA was reduced as well (from 2.8 ± 0.7 to 1.6 ± 0.4 for VAMP7 siRNA#1 and from 3.2 ± 0.2 to 0.9 ± 0.4 for VAMP7 siRNA#2, respectively; ^**^p < 0.01; Figure 5E; Video 4). We also used a second, independent assay to confirm our TIRF data. Because FACS-based degranulation assays measure not only CG fusion, but also LAMP1-containing lysosome fusion (26, 27), we used a recently developed, calcein-based killing assay (28). In excellent agreement with our TIRF data, we observed a significant reduction of killing efficiency in CTLs transfected with either VAMP7 siRNA (^***^p < 0.001; Figure 5F). In order to test the specificity of the effect of VAMP7 on CG fusion, we performed three control experiments. First, we transfected primary human CTLs with siRNA against VAMP4, which did not localize to CGs (Figures 2A–C), together with GzmB-mCherry and performed TIRFM. Although the knockdown of VAMP4 was very efficient (11.9 ± 4.7%; ^***^p < 0.001), we did not observe a reduction in either the percentage of CTLs showing CG fusion nor in the average number of CG fusion events per cell (Figure S3). Second, we analyzed the fusion of recycling endosomes, which usually precedes fusion of CGs at the IS (10), by co-transfecting CTLs with VAMP7 siRNA and Rab11-GFP, a marker for recycling endosomes. Again, we did not observe any difference in the percentage of CTLs showing recycling endosome fusion nor in the average number of recycling endosome fusion events per cell (Figure S4; Video 5). Third, in agreement with the lack of co-localization with LAT (Figures 2G–I), we found no decrease in the phosphorylation of the microtubule-associated protein (MAP) kinases Erk1 and Erk2 upon VAMP7 knockdown in Western blots with a phospho-specific antibody (Figure S5). We thus conclude that VAMP7 specifically mediates in the final fusion of CGs with the plasma membrane at the IS, without impairing LAT signalosome formation or recycling endosome fusion.

### VAMP7 Forms a SNARE Complex With Syntaxin11 and SNAP-23

**Figure 6:**
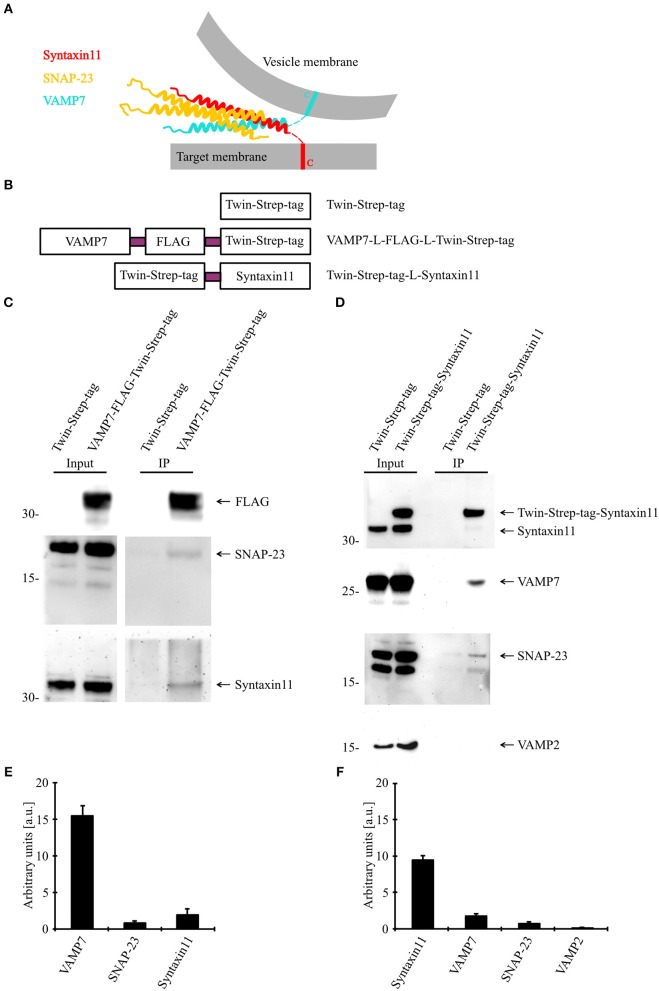
VAMP7 forms a SNARE complex with SNAP-23 and Syntaxin11. **(A)** Model illustrating the formation of SNARE complex between VAMP7 (v-SNARE), SNAP-23, and Syntaxin11 (t- SNARE) during cytotoxic granule fusion in human CD8+ T cells. **(B)** Different Twin-Strep-tag fusion constructs used for pulldown assay. Twin-Strep-tag was fused at the C terminus of VAMP7 and at the N terminus of Syntaxin11 with a (GGS)x3 linker. **(C)** Western blot of bead stimulated human CD8+ T cells transfected with Twin-Strep-tag-tagged VAMP7, immuno-precipitated with anti-FLAG antibody and detected with antibodies against FLAG, SNAP-23, and Syntaxin11. **(D)** Western blot of bead stimulated human CD8+ T cells transfected with Twin-Strep-tag-tagged Syntaxin11, immuno-precipitated with anti-Syntaxin antibody and detected with antibodies against Strep-tag, SNAP-23, VAMP7, and VAMP2. As control, cells were transfected with Twin-Strep-tag construct. Ten percentage of the lysates were loaded as input. **(E,F)** Densitometric quantification of the Western blots shown in **(C,D)**, respectively.

Because our data demonstrate an essential role for VAMP7 in the fusion of CGs and fusion processes are mediated by a SNARE complex consisting of vesicular (vSNARE) and target cell membrane SNAREs (tSNARE; Figure 6A), we sought to identify the corresponding SNARE partners of VAMP7. For that purpose, we generated fusion proteins for VAMP7 (VAMP7-Flag-Twin-Strep-tag) and Syntaxin11 (Twin-Strep-tag-Syntaxin11; Figure 6B) and transfected human CTLs with these constructs for an *in vitro* binding assay. Western blots of pull-downs from human CTLs expressing tagged VAMP7 revealed a specific band for both SNAP-23 and Syntaxin11, indicating them as the most likely tSNARE partners for VAMP7 (Figures 6C,E). Conversely, SNAP-23 and VAMP7 were pulled down from lysates of CTLs expressing tagged Syntaxin11 (Figures 6D,F). Importantly, other vSNAREs like VAMP2 were not detected in the pull-downs, demonstrating the specificity of the identified SNARE complex (Figure 6D). Together, these results define SNAP-23 and Syntaxin11 as tSNARE partners for VAMP7-mediated CG fusion in CTLs.

## Discussion

Our results identify a requirement for vSNARE VAMP7 in driving the fusion of CGs with the plasma membrane at the IS, thus playing a crucial role in mediating killing of target cells by CTLs in humans. This finding uncovers an important mechanistic difference in the release of cytotoxic substances between different species, because in primary CTLs from mouse this function is mediated by VAMP2 (11). A role of VAMP2 in human cytotoxic granule fusion could be clearly ruled out by the insensitivity to tetanus toxin (Figure 1), the lack of co-localization with granzyme B in activated and conjugated CTLs (Figure 2) and its reduced expression upon T cell activation (Figure S1).

In mice, VAMP7 is ubiquitously expressed and localizes to many intracellular organelles, most prominently to late endosomes and lysosomes. As a consequence, its interaction with various SNARE partners like Syntaxin1, Syntaxin3, Syntaxin4, Syntaxin7, Syntaxin8, SNAP-23, and SNAP-25 has been reported (29–32). In contrast to the “brevin” family of VAMPs (including VAMP2, VAMP3, and VAMP8), VAMP7 also contains an N-terminal longin domain that not only controls SNARE complex assembly through interaction with the coiled-coil domain (29, 33), but also determines VAMP7's subcellular localization through interaction with the clathrin adaptor AP-3, the guanine exchange factor Varp, and the endocytic protein Hrb (29, 34–36). Due to its diverse subcellular localizations, VAMP7 has been implicated in several membrane trafficking steps including late endosomes to lysosomes fusion, Golgi apparatus to plasma membrane exocytosis, or lysosomal and autophagosomal exocytosis (32, 37–41). Cellular processes involving VAMP7 range from cell polarization through apical transport, neuronal outgrowth, synaptic transmission, lysosomal secretion, membrane repair, cell migration, mitosis to phagocytosis (14, 42). Thus, a prediction on the role of VAMP7 in human CTLs based on the reported functions in very divergent cell types from mice is hardly possible. Rather, it appears indispensable to focus on reported roles of VAMP7 in immune cells.

In human immune cells, both VAMP7 and VAMP8 have been shown to form a SNARE complex with Syntaxin4 and SNAP-23 that mediates activation-induced degranulation of mast cells (31, 43). In human eosinophils and neutrophils, VAMP7 also interacts with Syntaxin4 and SNAP-23 to enable the release of mediators (44, 45). Finally, VAMP7 was shown to be involved in CG fusion in both human NK cells and in human NK-like cell lines (46, 47). VAMP7 co-localized, as did VAMP4, with perforin, and its down-regulation by siRNA led to a strong reduction in degranulation activity. However, VAMP4 down-regulation also led to a reduction in degranulation activity, and VAMP7 was also shown to be involved in cytokine release, arguing for a potentially indirect effect on CG fusion (47). Since these studies did not use methodology like TIRF microscopy to directly observe CG fusion in real-time, any role of these VAMP isoforms in the maturation of CGs could have caused the observed phenotype.

Thus, our data in primary, human CTLs presented here extend the available data on human immune cells and unambiguously identify the vSNARE mediating CG fusion at the IS. We not only find a high degree of co-localization of VAMP7 with cytotoxic granule markers granzyme B and perforin, but also demonstrate that VAMP7 and granzyme B fuse simultaneously at the IS. Concurrently, we could rule out a potential role of VAMP4 in CG release by both co-localization studies and CG fusion analysis through TIRFM. We also show that *in vitro* VAMP7 and SNAP-23 form a SNARE complex with Syntaxin11, and not Syntaxin4, for mediating CG fusion in primary human CTLs. Rather, Syntaxin4 appears to be involved in the early steps of IS formation of CTLs by regulating, in concert with VAMP8, the fusion of recycling endosomes with the plasma membrane (10, 48).

The presence of the longin domain in VAMP7 might prove helpful in fighting lethal diseases in humans like FHL and allow modification of the efficiency of CTL cytotoxicity. Formation of the SNARE complex for CG fusion, consisting of VAMP7, SNAP-23, and Syntaxin11, is an all-or-none reaction, most evident in lethal FHL4. In contrast, the regulatory longin domain in VAMP7 allows for a subtle interference of subcellular localization and endocytic efficiency. These manipulations would then allow to gradually modify cytotoxicity of human CTLs in a very subtle way and, at least theoretically, enable novel strategies for immunotherapy in pathophysiological settings.

In conclusion, we identify VAMP7 as the major vSNARE for mediating fusion of CGs in primary, human CTLs. Since this process is of outmost importance to protect our body against infections and tumors, VAMP7 might serve as a major target for the development of innovative drugs for immunotherapy in the future.

## Data Availability

All datasets generated for this study are included in the manuscript and/or the Supplementary Files.

## Ethics Statement

The local ethics committee (Ärztekammer des Saarlandes, 66002 Saarbrücken) has approved research with human material performed for this study.

## Author Contributions

PC, KR, DG, and MS performed the experiments. EK analyzed results and made the figures. YB and JR designed the research and wrote the paper.

### Conflict of Interest Statement

The authors declare that the research was conducted in the absence of any commercial or financial relationships that could be construed as a potential conflict of interest.
