# Supplementary material for: Cytotoxic Granule Exocytosis From Human Cytotoxic T Lymphocytes Is Mediated by VAMP7
Source: Front Immunol. 2019 Aug 7;10:1855. doi: 10.3389/fimmu.2019.01855 (PMC6692471; doi:10.3389/fimmu.2019.01855)
Supplement: Supplementary file 6 [file Data_Sheet_1.docx]

Supplementary Material

## Supplementary Figures

**
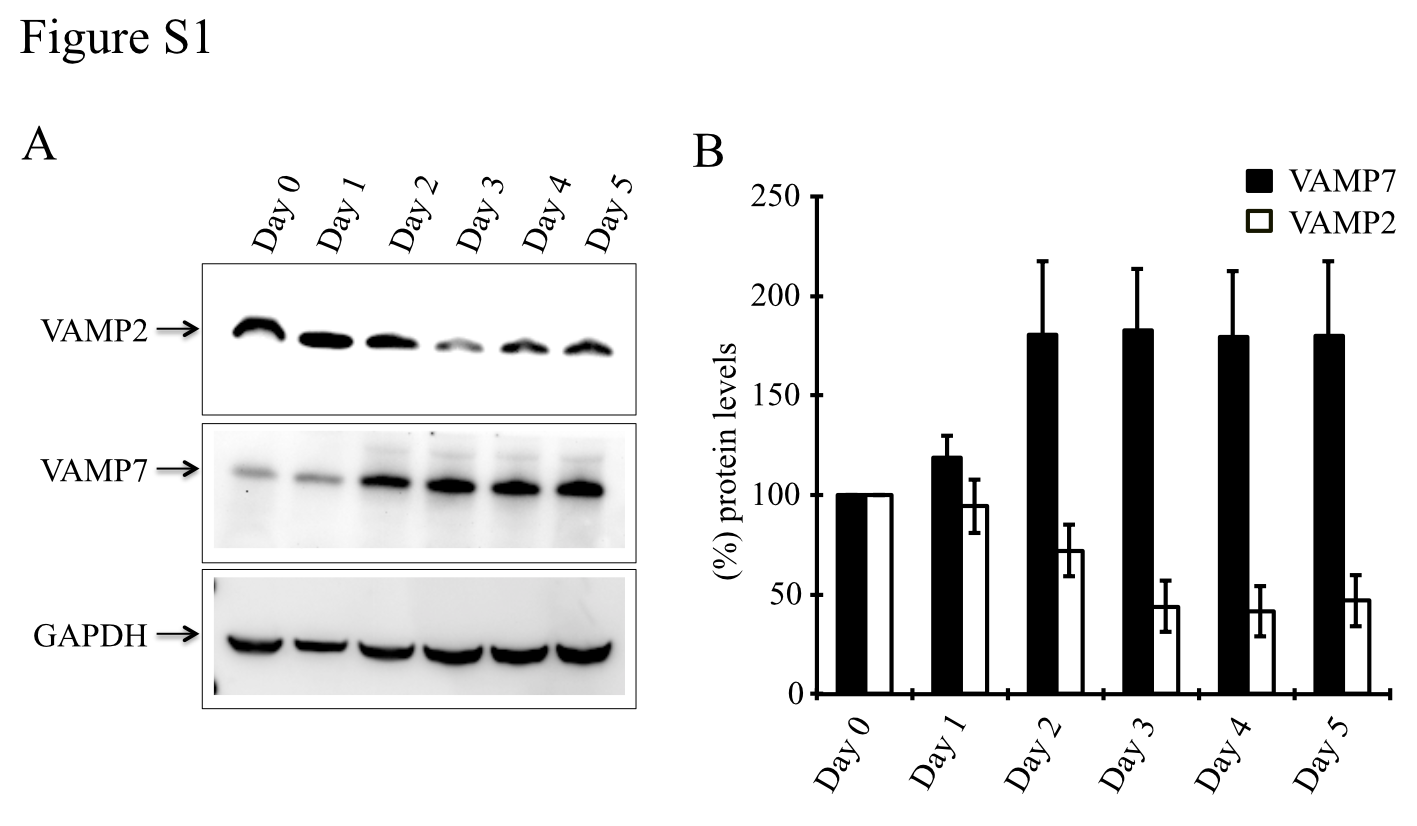
**

**Supplementary Figure 1.** **VAMP7 expression levels were upregulated upon activation whereas VAMP2 levels decreased over time.** (A) VAMP2 and VAMP7 expression in naive and bead-stimulated CD8+ T cells for the indicated days determined by Western blot analysis. (B) Graphs represent expression of VAMP2 and VAMP7 normalized to GAPDH. (N=3, *p*=0.222 (day 1), *p*=0.0505 (day 2), *p*=0.0142 (day 3), *p*=0.0178 (day 4) and *p*=0.0274 (day 5); (t-test)). Bars show mean ± SEM.

**
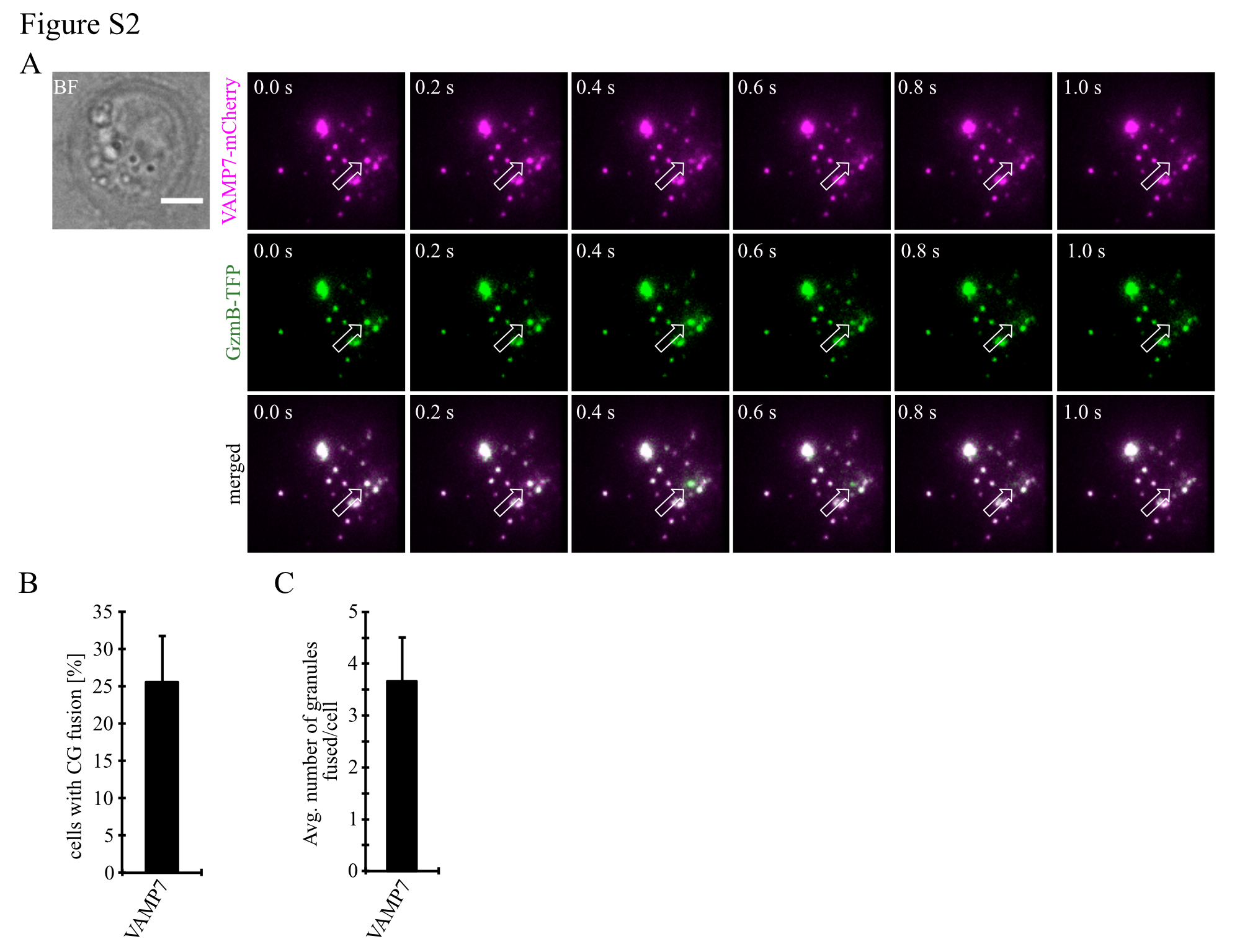
**

**Supplementary Figure 2.** **VAMP7 fuses at the plasma membrane.** (A) Bead stimulated human CD8+ T cells co-transfected with VAMP7-mCherry and granzyme B-TFP constructs and imaged 12 h after transfection. Selected live-cell TIRF microscopy images of VAMP7-mCherry and granzyme B-TFP in a transfected CTL in contact with an anti-CD3 coated coverslip. Fusion events are indicated with open arrowheads. (B) Mean percentage of cytotoxic granule fusion from three different donors (n=29). (C) Mean average number of granules fused over time in the TIRF plane per cell (n=29). Bars indicate ± SEMs. Scale bar, 5 µm.

**
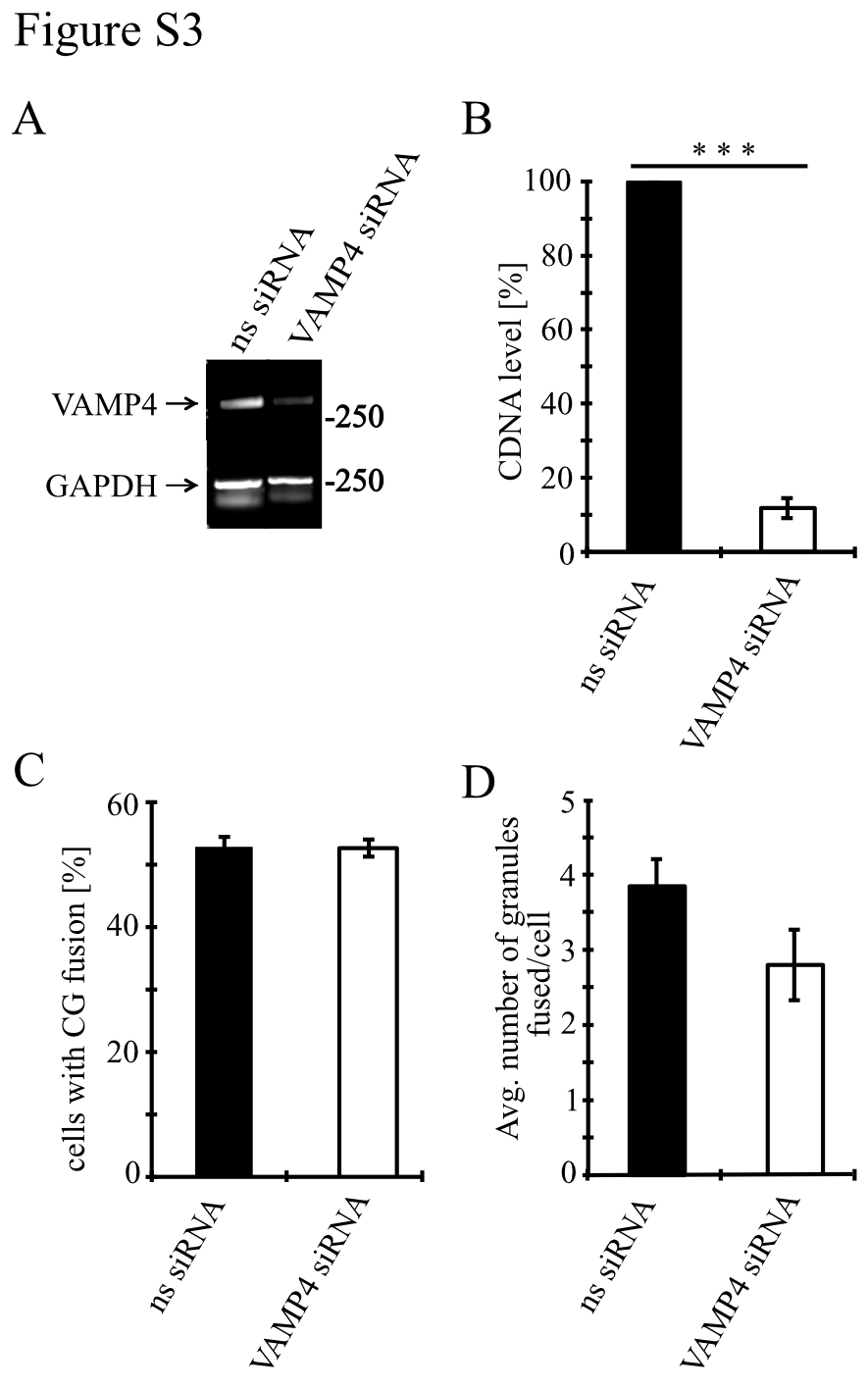
**

**Supplementary Figure 3.** **Knockdown of VAMP4 has no effect on fusion of cytotoxic granules at the IS.** (A) Bead-stimulated human CD8+ T cells transfected with ns-siRNA or siRNA against VAMP4 as indicated. Bands represent VAMP4 cDNA levels after 12 h of transfection by semi-quantitative PCR. (B) VAMP4 cDNA levels quantified by densitometry normalized to GAPDH from three independent experiments. Graphs represent means, normalized expression of VAMP4 (N=3, ****p* < 0.001; (t-test)). (C) Mean percentage of cytotoxic granules fusion in cells transfected with either ns-siRNA (n=82) or VAMP4-siRNA1 (n=53) (N=3, *p* = 0.997; (t-test)). (D) Mean average number of granules fused over time in the TIRF plane per cell for ns-siRNA (n=82) and VAMP4-siRNA1 (n=53) (N=3, *p* = 0.154; (t-test)). Bars indicate ± SEMs.

**
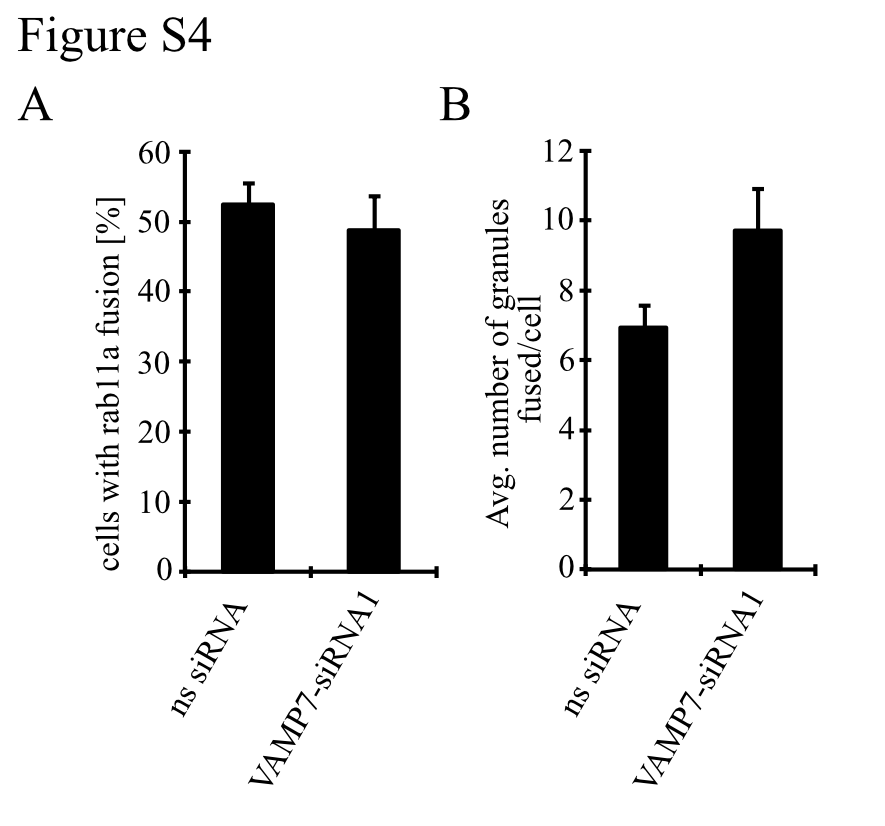
**

**Supplementary Figure 4.** **Rab11a fusion at the plasma membrane is unchanged upon VAMP7 knockdown.** (A) Bead stimulated human CD8+ T cells co-transfected with mCherry-Rab11a construct and either with ns-siRNA or VAMP7-siRNA1 and imaged 12 h after transfection. Mean percentage of cytotoxic granule fusion in cells transfected with VAMP7-mCherry from three different donors for ns-siRNA (n=44) and VAMP7-siRNA1 (n=46) (N=3, *p* = 0.558; (t-test)) (B) Mean average number of granules fused over time in the TIRF plane per cell for ns-siRNA (n=44) and VAMP7-siRNA1 (n=46) (N=3, *p* = 0.107; (t-test)).

**
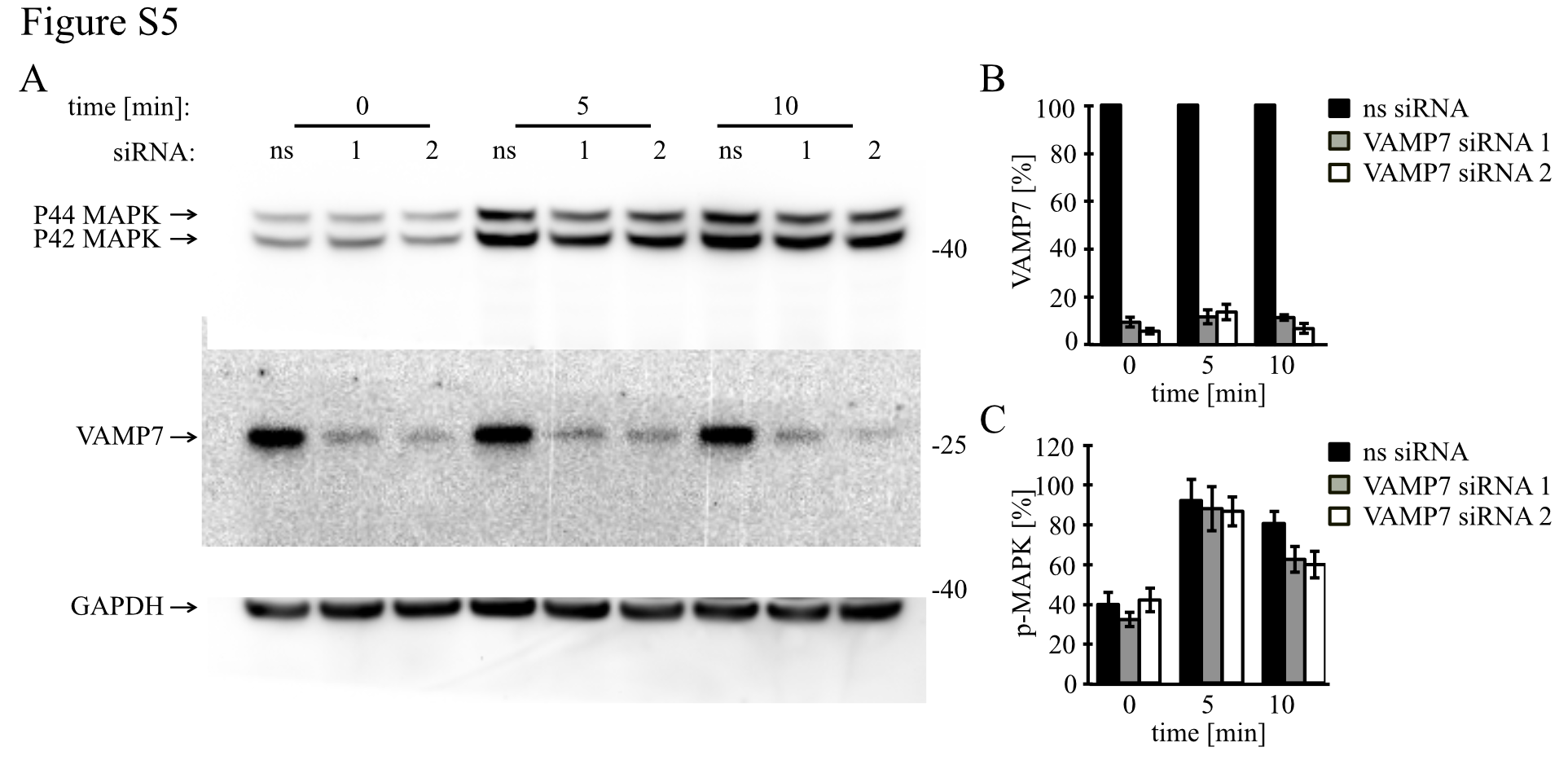
**

**Supplementary Figure 5.** **Phosphorylation of MAP kinases Erk1 and Erk2 is unaltered upon VAMP7 knockdown.** (A) Lysates from bead-stimulated human CTLs transfected with either control or siRNA1 or siRNA2 against VAMP7 and activated for 0, 5 or 10 min with mAb against CD3 and CD28 and blotted for p-MAPK (top), VAMP7 (middle) and GAPDH (bottom) as the loading control. (B) Quantification of VAMP7 protein expression (in % normalized to control siRNA-treated CTLs) performed by densitometry. Bars show mean ± SEM and data pooled from five independent experiments (siRNA1, N=5; ****p* < 0.001 and siRNA2, N=5; ****p* < 0.001 (t-test)). (C) The intensity of the signals for phosphorylated MAPK measured by immunoblot analysis, normalized to GAPDH and presented relative to that of unstimulated cells expressing control siRNA (N=5; 0 min, *p* = 0.417; 5 min, *p* = 0.651; 10 min, *p* = 0.088 (One-way ANOVA test). Bars show mean ± SEM.

## Legends for Supplementary Videos

**Video 1.** **Fusion of cytotoxic granules at the IS (TIRFM).** Real-time visualization of CGs fusing at the IS in CTLs monitored by TIRFM. CTLs were co-transfected with granzyme B-mCherry and TeNT-GFP or GFP alone. 490 frames were displayed at 28 frames/s. The corresponding still image is shown in Fig. 1 C. Scale bar, 5 µm.

**Video 2.** **Fusion of VAMP7 containing vesicles along with granzyme B (TIRFM).** Real-time visualization of CGs fusing at the IS from CTLs expressing VAMP7-pHuji and granzyme B-mTFP constructs. 120 frames were displayed at 15 frames/s. The corresponding still image is shown in Fig. 4 A. Scale bar, 5 µm.

**Video 3.** **Polarization and fusion of VAMP8-containing vesicles at the IS before CGs (TIRFM).** Real-time visualization of VAMP8-containing vesicles polarise and fuse at the IS before VAMP7-containing CGs. CTLs were co-transfected with VAMP7-mCherry and VAMP8-TFP constructs. 1200 frames were displayed at 40 frames/s. The corresponding still image is shown in Fig. 4 D. Scale bar, 5 µm.

**Video 4.** **Reduced fusion of cytotoxic granules at the IS from CTLs depleted for VAMP7 (TIRFM).** Real-time visualization of CGs fusing at the IS from CTLs expressing granzyme B-mCherry constructs. 497 frames were displayed at 30 frames/s. The corresponding still image is shown in Fig. 5 C. Scale bar, 5 µm.

**Video 5. Fusion of recycling endosomes at the IS (TIRFM).** Real-time visualization of recycling endosomes fusing at the IS in CTLs monitored by TIRFM. CTLs were co-transfected with ns-siRNA or VAMP7-siRNA1 along with mCherry-Rab11a. 3942 frames were displayed at 140 frames/s. The corresponding data is shown in Fig. S4 A & B. Scale bar, 5 µm.
